# Room temperature wafer bonding through conversion of polysilazane into $\hbox {SiO}_{2}$

**DOI:** 10.1038/s41598-024-51800-6

**Published:** 2024-01-13

**Authors:** Kai Takeuchi, Tadatomo Suga, Eiji Higurashi

**Affiliations:** 1 Graduate School of Engineering, Tohoku University, Sendai, 980-8579 Japan; 2 Collaborative Research Center, Meisei University, Tokyo, 191-8506 Japan

**Keywords:** Electrical and electronic engineering, Mechanical engineering

## Abstract

Room temperature wafer bonding is a desirable approach for the packaging and assembly of diverse electronic devices. The formation of $\hbox {SiO}_{2}$ layer at the bonding interface is crucial for a reliable wafer bonding as represented by conventional bonding techniques such as hydrophilic bonding and glass frit bonding. This paper reports a novel concept of room temperature wafer bonding based on the conversion of polysilazane to $\hbox {SiO}_{2}$ at the bonding interface. As polysilazane is converted to $\hbox {SiO}_{2}$ by hydrolysis, in this work, adsorbed water is introduced to the bonding interface by plasma treatment, thereby facilitating the formation of $\hbox {SiO}_{2}$ at the wafer bonding interface. The experimental results indicate that the adsorbed water from the plasma treatment diffuses into the polysilazane layer and facilitates its hydrolysis and conversion. The proposed method demonstrates the successful wafer bonding at room temperature with high bond strength without interfacial voids. This technique will provide a new approach of bonding wafers at room temperature for electronics packaging.

## Introduction

Wafer bonding technology has evolved significantly, particularly in the field of electronic applications manufactured at the wafer level to achieve high throughput. This advancement is especially critical for the packaging and assembly of MEMS devices, optical systems, and CMOS applications. Among these, wafer bonding of Si and Si-based materials is widely adapted for various applications including the packaging of MEMS sensors^1^, optical system sealing^2^, microchannel devices^3^, and 3D stacking of memory and logic devices^4^.

The formation of a $\hbox {SiO}_{2}$ layer at the bonding interface is the fundamental and highly effective approach for wafer bonding. One of the representing methods in this regard is hydrophilic bonding, where the Si surface containing physisorbed water and chemisorbed OH groups forms hydrogen and molecular bonds at the bonding interface^5,6^. In order to enhance the bonding quality and lower the process temperature, plasma treatments typically using $\hbox {O}_{2}$ or $\hbox {N}_{2}$ gas are widely employed to render Si bonding surfaces hydrophilic, facilitating water adsorption. Subsequent post-bonding annealing at 200-600 ${}^{\circ }\hbox {C}$ promotes Si oxidation at the bonding interface, thereby increasing the bond strength^7,8^.

In the field of MEMS packaging, glass frit bonding is also widely adapted through the formation of an $\hbox {SiO}_{2}$ interface using low-melting-point glass pastes^9,10^. Here, the bonding surfaces are coated with such glass pastes, and thermo compression bonding at 350-600 ${}^{\circ }\hbox {C}$ induces adhesion between the substrates through the reflowed glass layer. Hence, the formation of the $\hbox {SiO}_{2}$ layer at the bonding interface is pivotal for ensuring mechanical reliability.

However, the bonding process temperature is a crucial factor in industrial applications. Elevated temperatures can deteriorate device performance and the bonding interface’s reliability due to mismatches in the coefficient of thermal expansion between substrates. Consequently, low-temperature bonding, particularly at room temperature, is needed to accommodate next-generation electronics.

Regarding to the formation of $\hbox {SiO}_{2}$, the conversion of polysilazane into $\hbox {SiO}_{2}$ is a distinctive characteristic, represented by perhydropolysilazane (PHPS) $((\hbox {SiH}_{2}\hbox {NH})_{\textrm{n}})$, which undergoes hydrolysis when it interacts with water. This reaction leads to the formation of $\hbox {SiO}_{2}$, along with the release of byproducts such as $\hbox {NH}_{3}$ and $\hbox {H}_{2}$ ($\hbox {SiH}_{2}\hbox {NH}\,+\,2\hbox {H}_{2}\hbox {O}\,->\,\hbox {SiO}_{2}\,+\,\hbox {NH}_{3}\,+\,2\hbox {H}_{2}$)^11,12^.

PHPS can be converted into $\hbox {SiO}_{2}$ through various methods, including annealing within the temperature range of 200-1000${}^{\circ }\hbox {C}$^12,13^, infrared (IR) irradiation^14–16^, treatment with pH-controlled chemicals^17^, and exposure to moisture^18,19^. Since PHPS forms $\hbox {SiO}_{2}$ membrane by simple approaches compared to vacuum process such as chemical vapor deposition (CVD), PHPS is widely adapted to the coating of polymer films and metals^20–22^ and interlayer dielectrics^23,24^.

It is worth noting that the PHPS conversion into $\hbox {SiO}_{2}$ proceeds even at room temperature in the presence of water^11,13,25,26^. Hence, the conversion of PHPS to $\hbox {SiO}_{2}$ holds the potential for creating a bonding interface consisting of $\hbox {SiO}_{2}$ at room temperature, by introducing $\hbox {H}_{2}\hbox {O}$ at the PHPS interface, for instance, through the use of plasma hydrophilic treatment.

Based on this concept, this study proposes a novel approach of room temperature wafer bonding by forming $\hbox {SiO}_{2}$ bonding interface utilizing the PHPS conversion. The plasma hydrophilic treatment is applied to the Si wafer surface or the PHPS layer, thereby introducing adsorbed water to facilitate the PHPS conversion at the bonding interface. This is followed by wafer bonding via PHPS, leading to the formation of a $\hbox {SiO}_{2}$ bonding interface at room temperature.

To investigate the proposed wafer bonding process via PHPS, two approaches were explored. The first approach involved utilizing the PHPS layer as a source of water and oxygen for the conversion of the PHPS bonding interface. In this case, the PHPS layers on the bonding pair wafers were directly subjected to plasma hydrophilic treatment. Wafer bonding was then performed via both side PHPS layers on the both of bonding pair wafers. The effects of plasma treatment were examined by comparing conditions where both sides, one side, or none of the PHPS layers underwent plasma treatment.

In the second approach, plasma treatment was applied to the Si wafer surfaces. This scenario involved plasma hydrophilic treatment that facilitated water adsorption on the Si wafer surfaces, followed by the PHPS coating on the Si wafers. This implies that the PHPS receives water not from the ambient air but from the Si wafer surface. Subsequently, wafer bonding was performed via one side PHPS layer using the PHPS-coated wafer and the plasma-treated Si wafer. For comparison, wafer bonding was also performed via one side PHPS layer without plasma treatment to the wafer surface.

## Results

**Figure 1:**
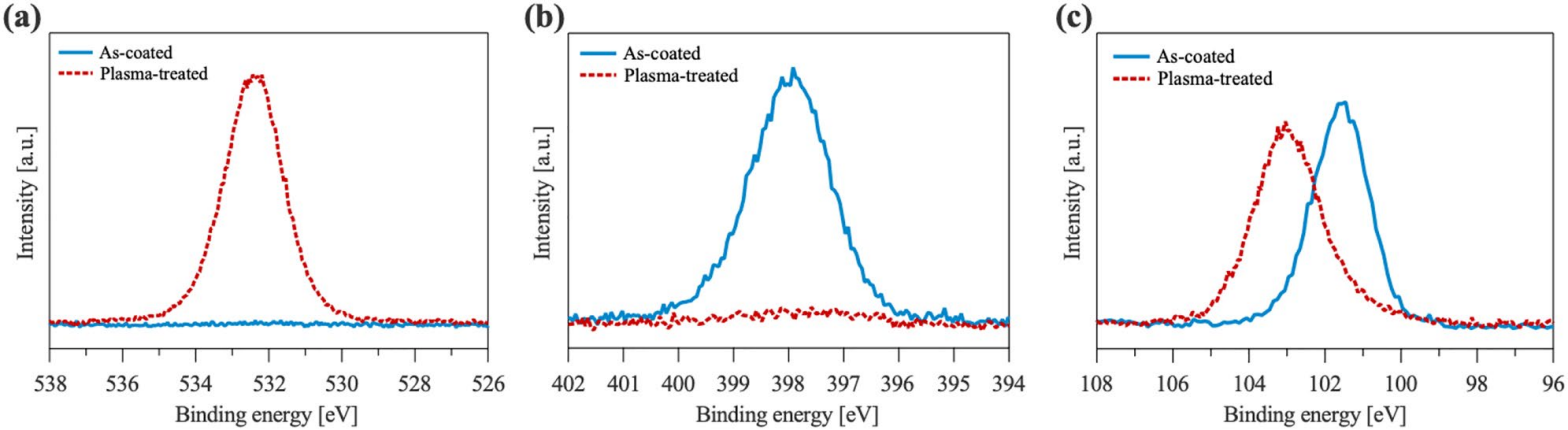
XPS core spectra of the PHPS surfaces before and after the $\hbox {N}_{2}$ plasma hydrophilic treatment for (**a**) O1s, (**b**) N1s, and (**c**) Si2p peaks.

In order to investigate the PHPS conversion into $\hbox {SiO}_{2}$ at room temperature by plasma hydrophilic treatment, chemical components of the PHPS coating on wafers were analyzed by x-ray photoelectron spectroscopy (XPS). Figure 1 presents the XPS core spectra of the PHPS surface before and after $\hbox {N}_{2}$ plasma treatment. When the PHPS layer is just coated, oxygen peak is not observed, accompanied by a significant peak of nitrogen. The silicon peak at around 101.3 eV indicates silicon nitride^27,28^. This is attributed to the intrinsic composition of PHPS^16,29,30^. However, when the PHPS layer is treated with plasma, an increase in the oxygen peak and a decrease in the nitrogen peak are significant. Moreover, the silicon peak shifts towards a higher binding energy around 103.5 eV, indicating the presence of silicon oxide^27,28^. Since the $\hbox {N}_{2}$ plasma itself does not contain $\hbox {H}_{2}\hbox {O}$ and $\hbox {O}_{2}$, it is suggested that the water adsorbs on the PHPS surface after the plasma treatment. Subsequently, the PHPS layer consumes the adsorbed water present on its hydrophilic surface for the conversion reaction.

**Figure 2:**
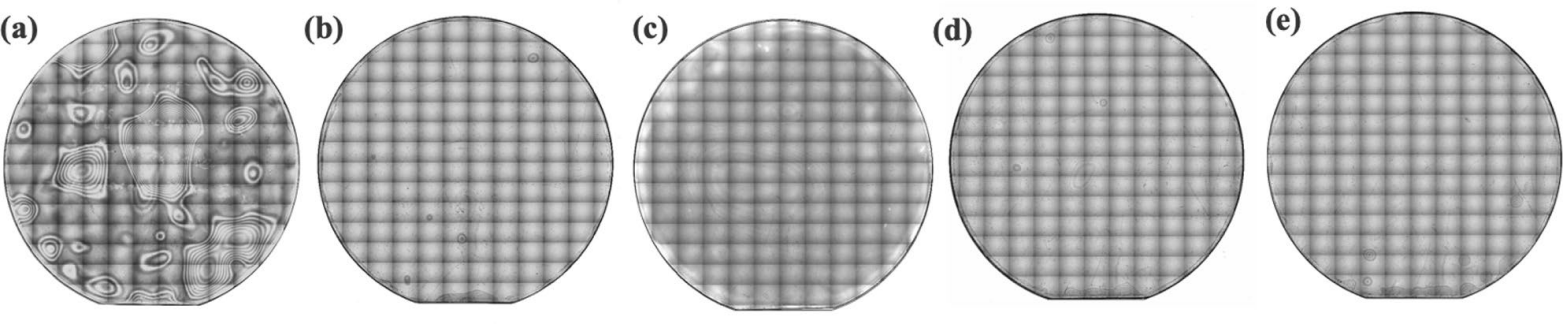
Stitched IR images of the bonded wafers via PHPS. (**a**) Bonding via both side PHPS layers with plasma treatment for both PHPS layers, (**b**) bonding via both side PHPS layers with plasma treatment for one PHPS layer, (**c**) bonding via both side PHPS layers without plasma treatment, (**d**) bonding via one side PHPS layers with plasma treatment, and (**e**) bonding via one side PHPS layers without plasma treatment.

The stitched IR images of the typical bonded wafers with PHPS layers are depicted in Fig. 2. These images reveal the presence of interfacial voids, which appear as patterns with interference fringes. Basically the bonding interface shows a good adhesion without significant interfacial voids except the bonding via both side PHPS layer with both side plasma treatment (Fig. 2 (**a**)). Due to the high viscosity of PHPS before its conversion to $\hbox {SiO}_{2}$, good adhesion is achieved through the PHPS layers. Additionally, it is indicated that submicron-sized particles can become embedded within the soft PHPS layers^17,31^.

Conversely, bonding via both sides plasma-treated PHPS layers results in large interfacial voids, as shown in Fig. 2 (**a**). As indicated by the XPS analysis, plasma treatment converts the PHPS surface to hard $\hbox {SiO}_{2}$. However, since the spin-coated PHPS layer does not possess a smooth and flat surface, the converted $\hbox {SiO}_{2}$ surface does not fully compensate for surface asperities, leading to lower quality adhesion.

**Figure 3:**
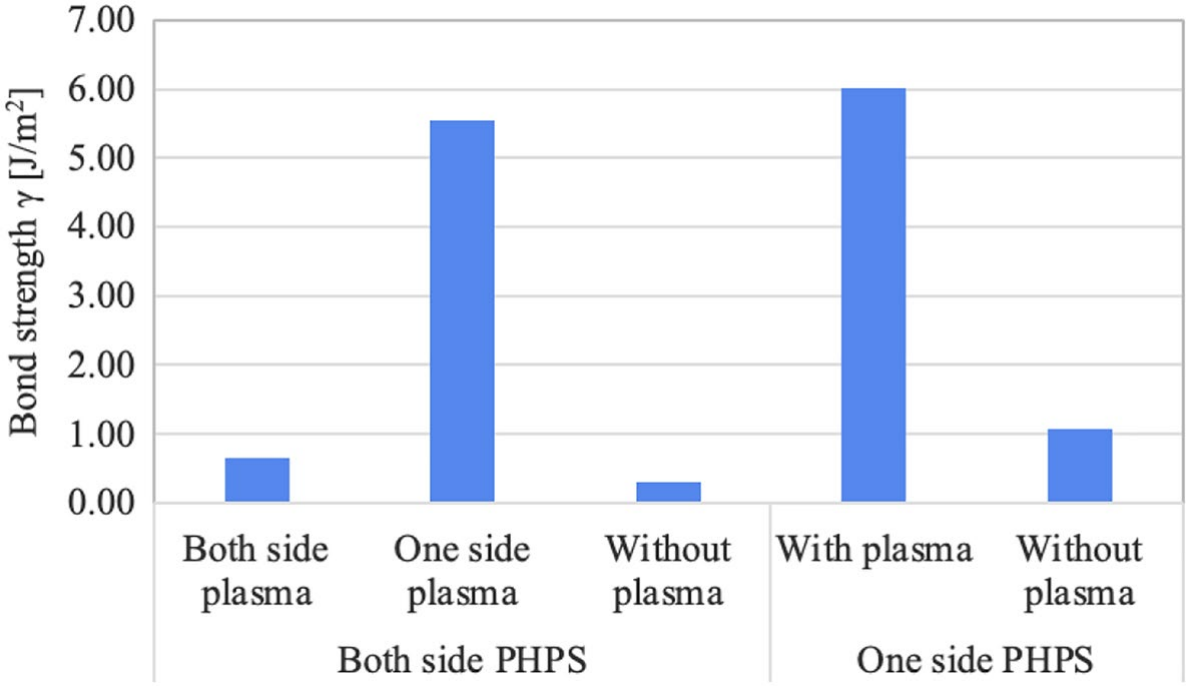
Bond strength $\gamma$ of the bonded wafers via PHPS layers at room temperature.

The results of the bond strength measurements obtained through blade insertion tests are presented in Fig. 3. For the bonding via both side PHPS layers, the bond strength is low when both PHPS layers are subjected to plasma treatment or when plasma treatment is not performed, yielding bond strengths of 0.64 J/m$^2$ and 0.30 J/m$^2$, respectively. In the scenario where both sides of the PHPS layers undergo plasma treatment, both surfaces are converted into $\hbox {SiO}_{2}$ with surface asperities. Consequently, weak adhesion and a low bond strength result, which aligns with the results of the IR observations. In cases where plasma treatment is not performed, an insufficient supply of water at the bonding interface prevents the PHPS layers from conversion, resulting in a low bond strength.

Conversely, when only one side of the PHPS layers is treated with plasma, a significantly improved bond strength of 5.54 J/m$^2$ is achieved. In this case, the bonding interface features the adhesive PHPS layer in contact with the hydrophilic PHPS layer. The hydrophilic layer with adsorbed water facilitates the conversion of the untreated PHPS layer into $\hbox {SiO}_{2}$. As a result, both strong adhesion and high bond strength are attained.

In the cases of the bondings via the single side PHPS layer, when wafer bonding is conducted without the plasma hydrophilic treatment, the resulting bond strength measures 1.07 J/m$^2$. In contrast, bonding with plasma treatment exhibits a significantly improved bond strength of 6.02 J/m$^2$. Consistent with the bonding using both side PHPS layers, the plasma treatment introduces adsorbed water to the PHPS layer, thereby promoting its conversion to a mechanically stable $\hbox {SiO}_{2}$ interface at room temperature.

**Figure 4:**
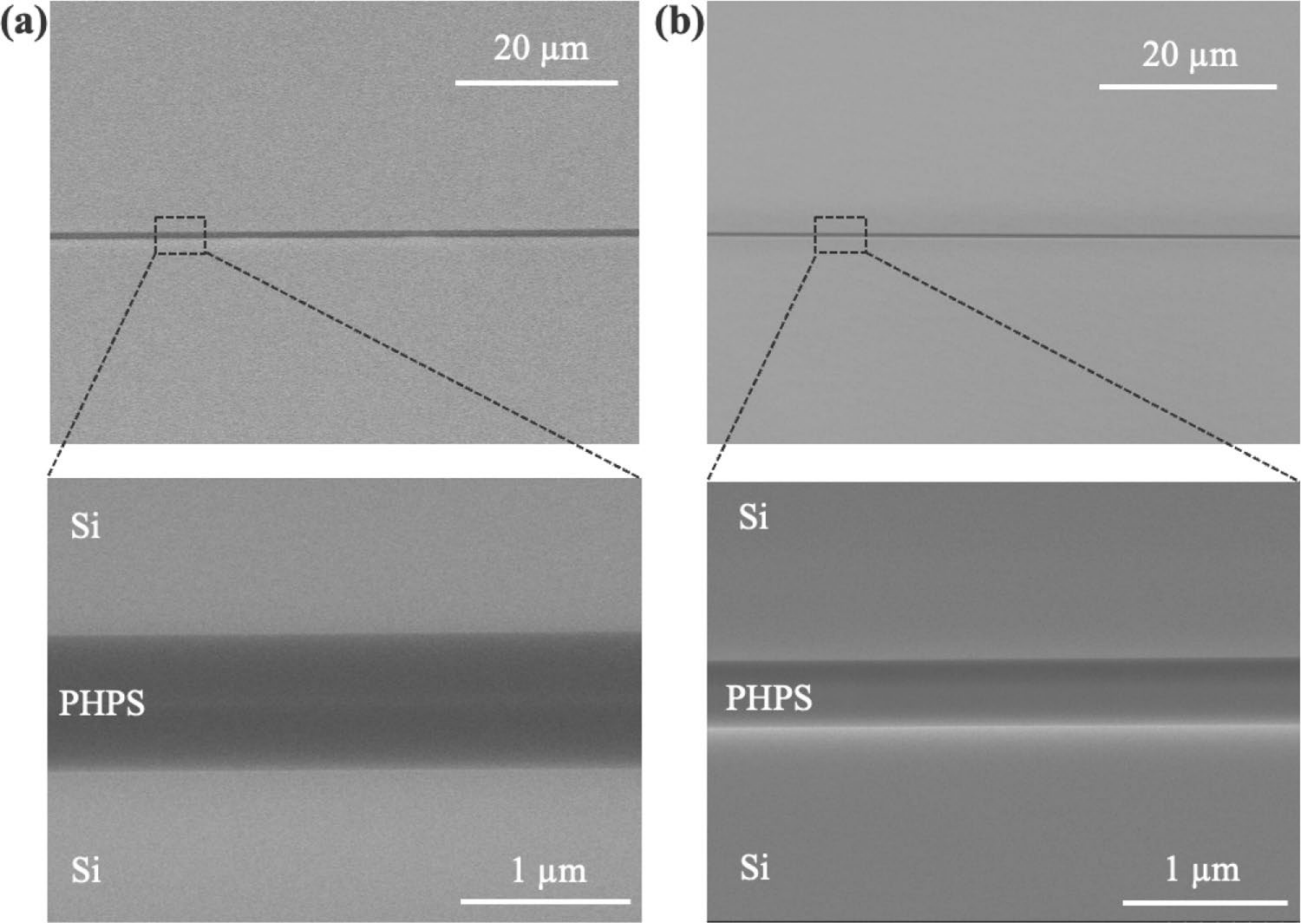
Cross-sectional SEM observation of the bonding interface of (**a**) both side PHPS layers with one side plasma treatment and (**b**) one side PHPS layer with plasma treatment.

Figure 4(a) shows the cross-sectional scanning electron microscopic (SEM) images of the bonding interface for the Si wafers bonded using PHPS layers on both sides with one side subjected to plasma treatment. In the low magnification image, the bonding interface appears uniform and devoid of voids. At higher magnification, the bonding interface is seen to consist of PHPS layers, each with a thickness of 0.4 $\upmu$m, resulting in a total thickness of 0.8 $\upmu$m. For the bonding via single side PHPS layer with plasma treatment shown in Fig. 4(b), a uniform and void-free bonding interface is also clearly visible, with the thickness of the PHPS layer measuring 0.4 $\upmu$m, consistent with the thickness observed in the bonding using double side PHPS layers.

**Figure 5:**
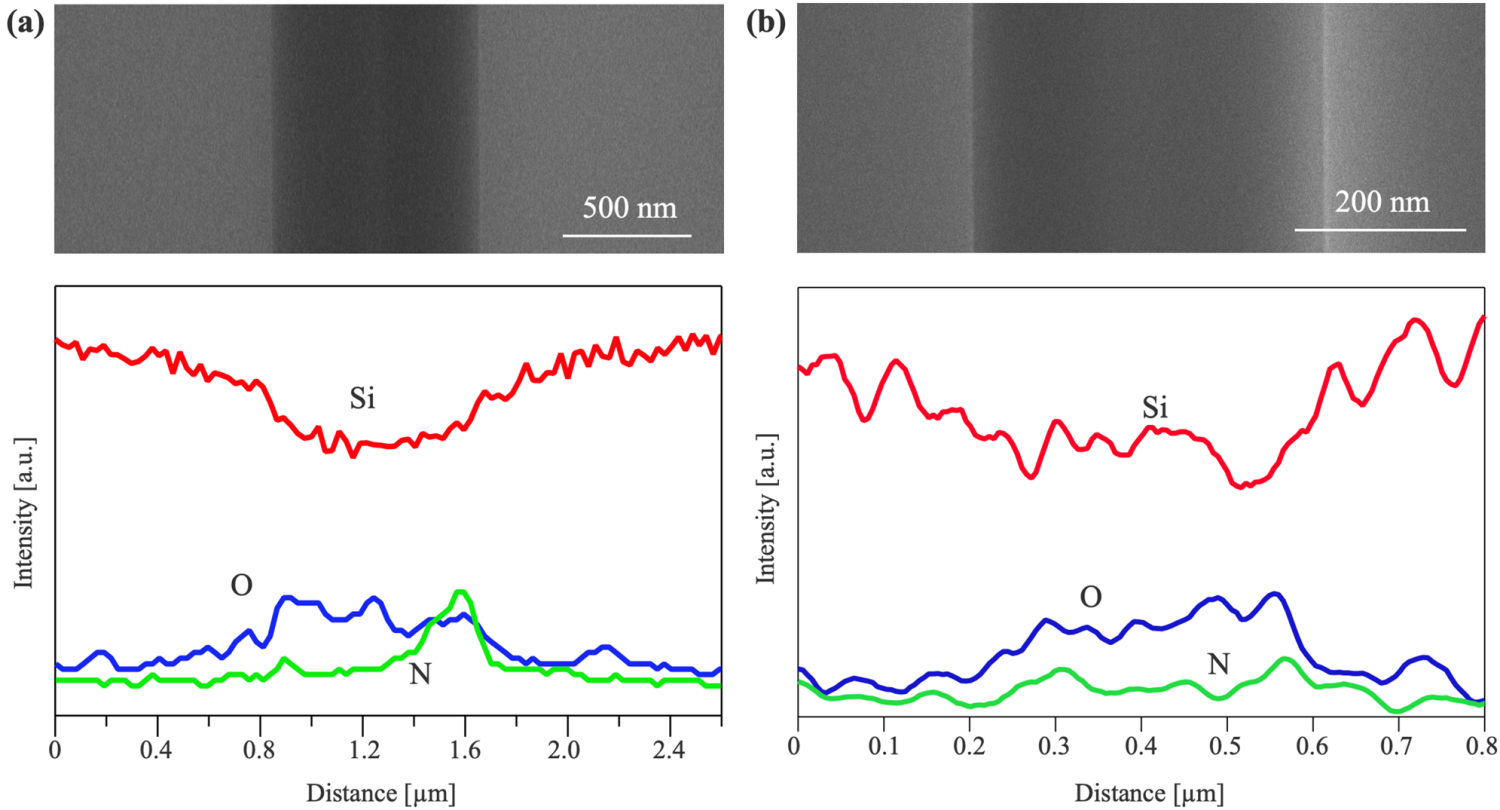
EDX line profile across the bonding interface via (**a**) both side PHPS layers with one side plasma treatment and (**b**) single side PHPS layer with plasma treatment.

Energy dispersive x-ray spectroscopy (EDX) analysis was performed across the bonding interface, and the results are presented in Fig. 5. For the bonding via double side PHPS layers with one side plasma, the presence of O and N is detected at the interface of the PHPS layers, while the Si signal is relatively lower compared to the bulk Si area. This can be attributed to the lower density of Si atoms in the converted PHPS layers. Moreover, the O intensity appears to be uniformly distributed across the PHPS layers, while the N intensity is relatively weak on the left side of the PHPS layers but as strong as O on the right side.

This distribution of N intensity is attributed to the differing treatment of the two PHPS layers. Specifically, the left side PHPS layer in Fig. 5(a) is treated with plasma, while the right side is not. The dominant O signal on the left side is consistent with the PHPS conversion by plasma treatment, while the strong N signal on the right side suggests that the PHPS layer in this region is partially converted to $\hbox {SiO}_{2}$.

The EDX line profile across the bonding interface of the single PHPS layer is illustrated in Fig. 5(b). At the PHPS layer area, the Si intensity is lower, while the presence of O and N is detected at the bonding interface. In comparison to the bonding utilizing both side PHPS layers as shown in Fig. 5(a), the composition of the PHPS layer appears to distribute more uniformly across the bonding interface, and the N intensity is closer to the background level. This suggests a more uniform conversion of the PHPS layer to $\hbox {SiO}_{2}$ than the bonding via both side PHPS layers. Given that the single PHPS layer at the bonding interface is situated between hydrophilic Si surfaces, the adsorbed water diffuses into the PHPS layer from both sides. As a result, the conversion of PHPS to $\hbox {SiO}_{2}$ proceeds more uniformly than the bonding via both sides PHPS layers.

Furthermore, the N signal in EDX can be partly attributed to the byproducts of $\hbox {NH}_{3}$ resulting from the PHPS conversion. Given that N is generally spread throughout the PHPS layers, it suggests the diffusion of $\hbox {NH}_{3}$ and $\hbox {H}_{2}$ byproducts into the PHPS layer. As the converted PHPS into $\hbox {SiO}_{2}$ has an amorphous structure, both $\hbox {NH}_{3}$ and $\hbox {H}_{2}$ are expected to diffuse similarly to $\hbox {H}_{2}\hbox {O}$.

**Figure 6:**
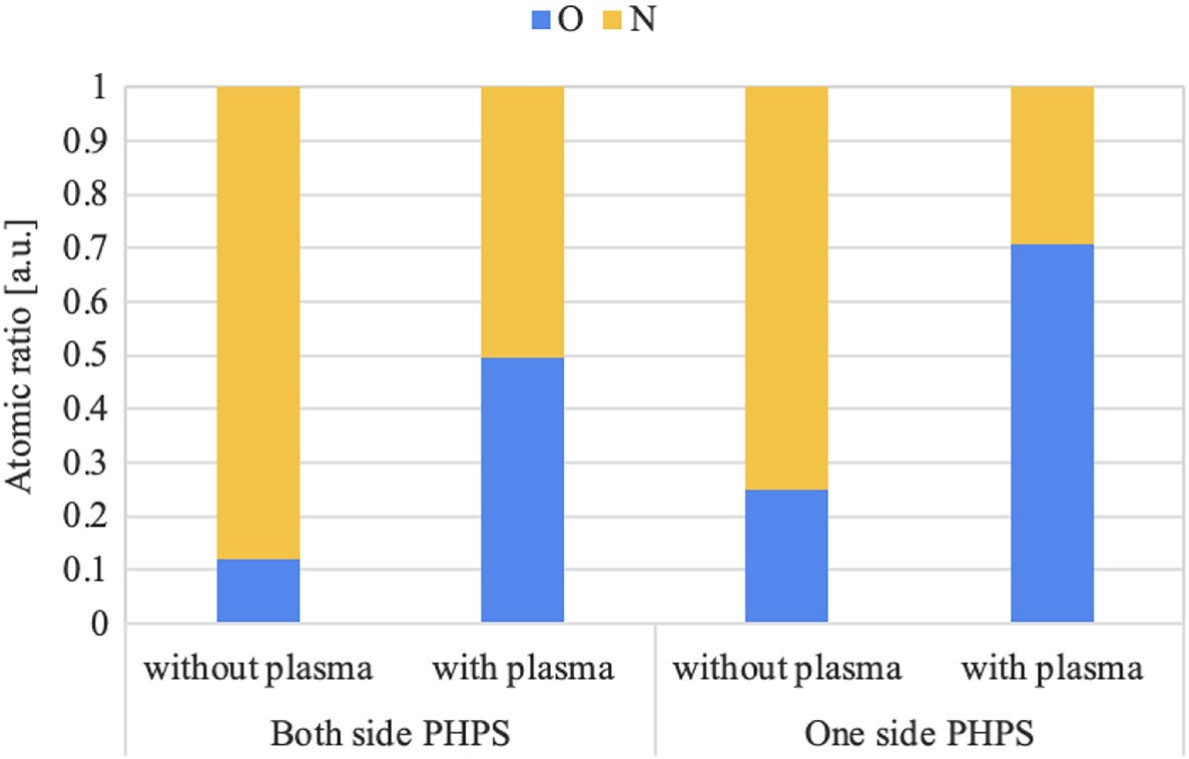
Atomic ratio of O and N on the debonded wafer surface of the bonding via both and one side PHPS layers with and without plasma treatment.

To investigate the PHPS conversion at the bonding interface, XPS analysis was performed on the debonded surface for both the bondings using both side PHPS layers and a single side PHPS layer, with and without plasma treatment. The atomic ratio of O and N was calculated from the O1s and N1s peaks for each condition, as illustrated in Fig. 6.

For bonding via both side PHPS layers without plasma treatment, the N ratio was 0.88, suggesting that the N in the PHPS molecules was slightly replaced by O from the natural adsorption of water on the Si wafers^32^. However, when one of the both side PHPS layers was treated with plasma, the N ratio decreased to 0.50, indicating that the adsorbed water from the plasma treatment replaced the N at the bonding interface.

On the other hand, the debonded surface with a single side PHPS layer also exhibited a high N ratio of 0.75 without plasma treatment. Similarly to bonding via both side PHPS layers, plasma treatment reduced the N ratio to 0.30 for bonding via a single side PHPS layer. As indicated by the cross-sectional EDX analysis, the single PHPS layer is sandwiched between the hydrophilic Si surfaces. The conversion of the single side PHPS layer proceeded further due to the relatively larger amount of water present at the bonding interface, as the amount of PHPS was half of that in the bonding via both side PHPS layers. Consequently, the replacement of N by O proceeded more largely compared to the bonding via double side PHPS layers.

## Discussion

**Figure 7:**
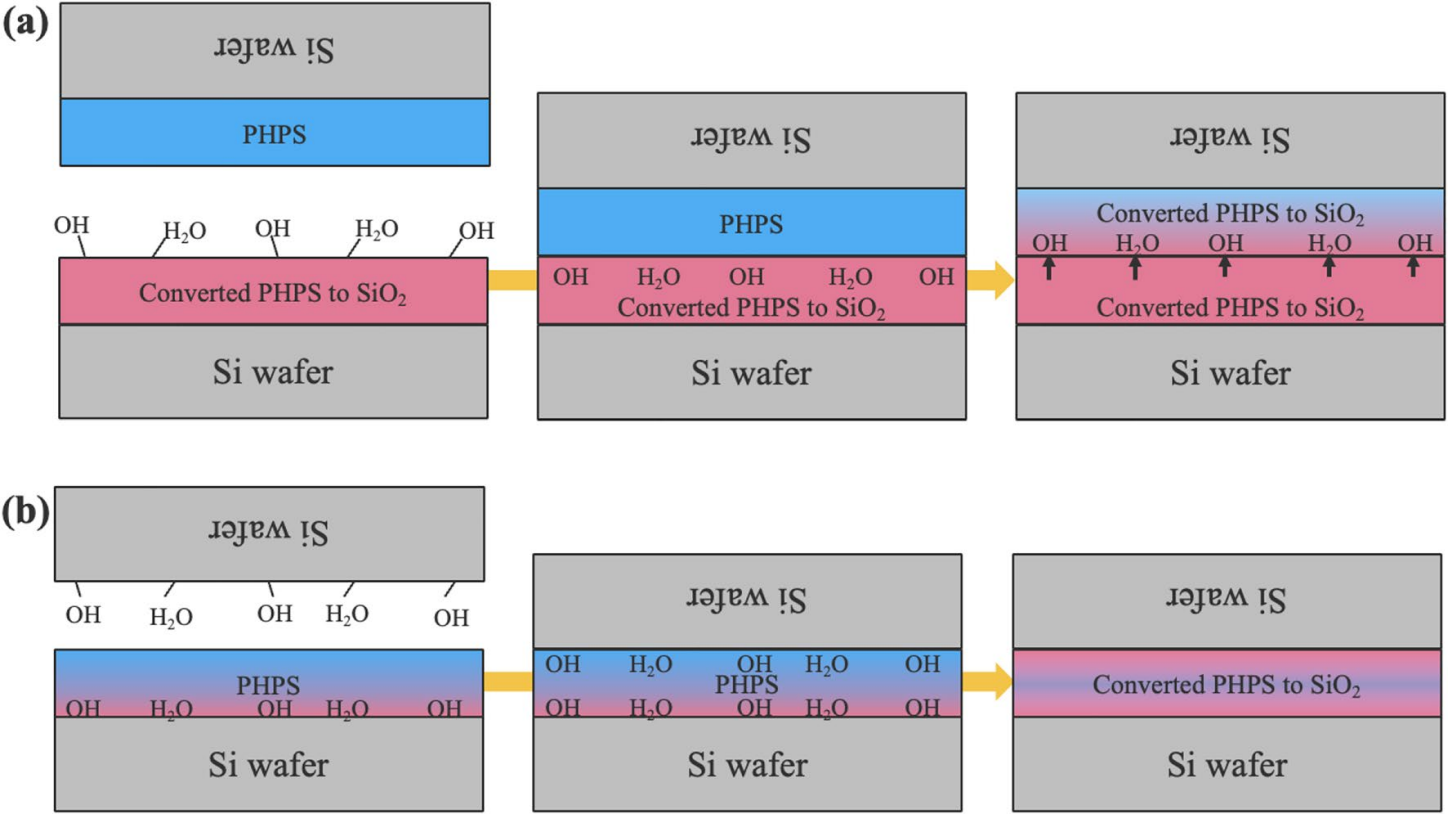
The room temperature wafer bonding mechanism via (**a**) both side PHPS layers with plasma treatment for one side PHPS layer and (**b**) one side PHPS layer with plasma treatment.

Based on the experimental results, we propose a room temperature bonding mechanism as illustrated in Fig. 7. For bonding with both-side PHPS layers, the plasma treatment initiates the conversion of one PHPS layer to $\hbox {SiO}_{2}$, facilitated by adsorbed water, as confirmed by the XPS analysis. Following the bonding of the plasma-treated PHPS layer with the untreated PHPS layer, the latter adheres to the substrates due to its ability to deform and compensate for surface asperities, as evidenced by IR imaging of the bonding interface. Subsequently, the water from the plasma-treated PHPS layer diffuses into the untreated PHPS layer, proceeding the hydrolysis of the untreated PHPS layer^29^. As the condensation polymerization of Si-OH groups proceeds and enhances the bond strength even at room temperature^6,33,34^, $\hbox {SiO}_{2}$ is formed at the bonding interface proceeds, resulting in a increased bond strength. The plasma-treated PHPS layer serves as a source of water and oxygen, supporting this conversion process, as indicated by cross-sectional EDX analysis.

In the case of bonding with a single PHPS layer, the PHPS layer is coated on the hydrophilic surface with adsorbed water, thus facilitating PHPS conversion including hydrolysis step starting from the rear side. It has also been reported that the adsorbed water on the substrates react with the PHPS layer coated on the substrate^32^. This conversion is driven by the diffusion of the adsorbed water originating from the Si surface into the PHPS layer. Concurrently, the PHPS layer serves as an adhesive during the bonding of the PHPS-coated wafer to the uncoated wafer. Given that the surface of the uncoated wafer is also hydrophilic due to adsorbed water, water similarly diffuses into the PHPS layer from the front side. Consequently, the PHPS layer, situated between two hydrophilic Si surfaces, undergoes more uniform conversion into $\hbox {SiO}_{2}$ compared to bonding via PHPS layers on both sides, as supported by the EDX analysis.

Compared to other wafer bonding techniques, the wafer bonding via PHPS is comparable to water glass bonding^35–37^ and sol-gel bonding^38–40^. Both of these techniques involve bonding through a liquid precursor of $\hbox {SiO}_{2}$, followed by an annealing step to create the bonding interface composed of $\hbox {SiO}_{2}$. However, a fundamental distinction lies in the mechanism of transformation to $\hbox {SiO}_{2}$. In sol-gel or water glass bonding, the conversion to $\hbox {SiO}_{2}$ occurs through condensation polymerization, necessitating annealing to eliminate $\hbox {H}_{2}\hbox {O}$ from the bonding interface. In contrast, the proposed method utilizing a PHPS layer achieves the formation of the $\hbox {SiO}_{2}$ bonding interface through hydrolysis, allowing for bonding at room temperature without the need for annealing.

Regarding to the conventional direct bonding of Si or $\hbox {SiO}_{2}$, wafer bonding via PHPS exhibits a comparable bonding quality. In conventional hydrophilic bonding of Si or $\hbox {SiO}_{2}$ with plasma hydrophilic treatment, the bond strength typically increases with post-bonding annealing. For Si/Si and $\hbox {SiO}_{2}$/$\hbox {SiO}_{2}$ wafer bonding, the typical bond strength $\upgamma$ is less than 1 J/m$^2$ without post-bonding annealing^6,41^. The bond strength $\upgamma$ increases to over 2 J/m$^2$ after annealing at temperatures no lower than 300 ${}^{\circ }\hbox {C}$. Despite the conventional hydrophilic wafer bonding requiring a heating step for a robust bonding interface, wafer bonding via PHPS achieves a high bond strength without annealing.

In addition, conventional hydrophilic wafer bonding is often associated with concerns about bubble formation at the bonding interface. Post-bonding annealing in hydrophilic bonding facilitates the condensation polymerization of Si-OH at the bonding interface. This process generates byproducts such as $\hbox {H}_{2}$ and residual $\hbox {H}_{2}\hbox {O}$, resulting in the formation of bubbles and voids at the bonding interface^41,42^. In contrast, wafer bonding via PHPS, as depicted in Fig. 2, does not exhibit the formation of bubbles, contributing to an improved bonding quality.

## Conclusions

In this paper, we investigated and demonstrated wafer bonding via PHPS at room temperature. Through the strategic use of plasma hydrophilic treatment on either the Si surface or the PHPS layer, we have effectively facilitated the conversion of PHPS into a robust bonding interface composed of $\hbox {SiO}_{2}$. This approach achieves the bond strength exceeding 5 J/m$^2$ and the void-free bonding interfaces, which are attributed to the combined effects of PHPS adhesion and $\hbox {SiO}_{2}$ formation. The XPS and EDX analysis also suggest the bonding mechanism that the adsorbed water by hydrophilic plasma treatment diffuses into the PHPS layer which results in the partial conversion of PHPS into $\hbox {SiO}_{2}$, contributing to the robust bonding interface. This technique will provide a new approach for the wafer bonding at room temperature for electronics packaging.

## Methods

4 inch Si wafers, with a thickness of 525 $\upmu$m, were utilized for the wafer bonding experiments. A 20 wt% solution of PHPS in dibutyl ether solvent was purchased.

In the case of the wafer bonding via both side PHPS layers, first, the wafers were coated with PHPS solution by spin coating at 2000 rpm for 20 s. The coated PHPS layers underwent a baking process on a hot plate at 100 ${}^{\circ }\hbox {C}$ for 5 min to volatilize the solvent. This baking step completely removes the solvent from the PHPS layer^32,43^, that is also supported by XPS and EDX analysis (data not shown). Sequentially, the PHPS layers were treated with $\hbox {N}_{2}$ plasma with 150 W power for 60 s to induce hydrophilicity on the PHPS layer’s surface. After the surface treatment, the PHPS-coated surfaces of the Si wafers were manually brought into contact under ambient air conditions at room temperature. For comparison, wafer bonding was also conducted with plasma treatment applied to both sides of the PHPS layers, one side of the PHPS layers, and without any plasma treatment.

In the case of the wafer bonding via one side PHPS layer, one wafer of a bonding pair was coated with PHPS. First, the bonding pair of Si wafers were treated with $\hbox {O}_{2}$ plasma followed by $\hbox {N}_{2}$ plasma to enhance water adsorption^44,45^, with both treatments conducted at a power of 150 W for 60 s each. After the plasma treatment, the wafers underwent a DI water rinse and were subsequently spin-dried. Following these surface preparations, the PHPS solution was spin-coated onto the one of the treated Si wafer pair, and solvent volatilization was conducted under the same conditions as described earlier. Finally, the Si wafer coated with PHPS and the uncoated wafer were bonded together in the ambient air at room temperature. The wafer bonding was also performed without the plasma hydrophilic treatment of Si wafers for comparison.

The PHPS layer underwent analysis using XPS to investigate surface compositions. The bonding quality was evaluated by IR imaging of the bonded wafers and bond strength measurements using the blade test^46^. The properties of the cross-sectional bonding interface were observed using SEM with EDX.

## Data Availability

The datasets used and/or analyzed during the current study available from the corresponding author on reasonable request.
